# The Association Between Breastfeeding and Growth Among Infants with Moderately Low Birth Weight: A Prospective Cohort Study

**DOI:** 10.1016/j.jpeds.2024.114003

**Published:** 2024-06

**Authors:** Krysten North, Katherine E.A. Semrau, Roopa M. Bellad, Sangappa M. Dhaded, Leena Das, Jnanindranath N. Behera, Irving Hoffman, Tisungane Mvalo, Rodrick Kisenge, Christopher R. Sudfeld, Sarah Somji, Rana R. Mokhtar, Linda Vesel, Shivaprasad Goudar, Sunil S. Vernekar, E.S. Siddhartha, Bipsa Singh, M.B. Koujalagi, Sanghamitra Panda, Eddah Kafansiyanji, Naomie Nyirenda, Melda Phiri, Friday Saidi, Nahya S. Masoud, Robert Moshiro, Danielle E. Tuller, Kiersten Israel-Ballard, Christopher P. Duggan, Anne C.C. Lee, Kimberly L. Mansen, Melissa F. Young, Karim Manji, LIFE study team

**Affiliations:** 1 Department of Pediatrics, Brigham and Women's Hospital, Boston, MA; 2 Harvard Medical School, Boston, MA; 3 Ariadne Labs at Brigham and Women's Hospital and the Harvard T.H. Chan School of Public Health, Boston, MA; 4 Department of Pediatrics, Jawaharlal Nehru Medical College, KLE Academy of Higher Education and Research, Belgaum, Karnataka, India; 5 Department of Pediatrics, Postgraduate Institute of Medical Education & Research and Capital Hospital, Bhubaneswar, Odisha, India; 6 Department of Pediatrics, S.C.B. Medical College, Cuttack, Odisha, India; 7 University of North Carolina Project Malawi, Lilongwe, Malawi; 8 Department of Medicine, Institute of Global Health and Infectious Diseases, School of Medicine, University of North Carolina at Chapel Hill, NC; 9 Department of Pediatrics, School of Medicine, University of North Carolina at Chapel Hill, NC; 10 Department of Paediatrics and Child Health, Muhimbili University of Health and Allied Sciences (MUHAS), Dar es Salaam, Tanzania; 11 Departments of Global Health and Population and Nutrition, Harvard T.H. Chan School of Public Health, Boston, MA; 12 Department of Paediatrics, J J M Medical College, Davangere, Karnataka, India; 13 Department of Paediatrics, Shri Jagannath Medical College and Hospital, Puri, Odisha, India; 14 Department of Paediatrics, City Hospital, Cuttack, Orissa, India; 15 Integrated Maternal and Child Health and Development, PATH, Seattle, WA; 16 Division of Gastroenterology, Hepatology, and Nutrition, Center for Nutrition, Boston Children's Hospital, and Department of Nutrition, Harvard TH Chan School of Public Health, Boston, MA; 17 Hubert Department of Global Health, Emory University School of Public Health, Atlanta, GA

**Keywords:** low birth weight, lactation support, infant feeding, breastfeeding, preterm, PIBBS, LATCH, small-for-gestational age, infant growth

## Abstract

**Objective:**

To assess the association between breastfeeding competency, as determined by Latch, Audible swallowing, Type of nipple, Comfort, and Hold (LATCH) and Preterm Infant Breastfeeding Behavior Scale (PIBBS) scores, and exclusive breastfeeding and growth among infants with low birth weight (LBW) in India, Malawi, and Tanzania.

**Study design:**

We conducted LATCH and PIBBS assessments among mother-infant dyads enrolled in the Low Birthweight Infant Feeding Exploration (LIFE) observational study of infants with moderately LBW (1500g-2499 g) in India, Malawi, and Tanzania. We analyzed feeding and growth patterns among this cohort.

**Results:**

We observed 988 infants. We found no association between LATCH or PIBBS scores and rates of exclusive breastfeeding at 4 or 6 months. Higher week 1 LATCH and PIBBS scores were associated with increased likelihood of regaining birth weight by 2 weeks of age [LATCH: aRR 1.42 (95% CI 1.15, 1.76); PIBBS: aRR 1.15 (95% CI 1.07, 1.23); adjusted for maternal age, parity, education, residence, delivery mode, LBW type, number of offspring, and site]. Higher PIBBS scores at 1 week were associated with improved weight gain velocity (weight-for-age z-score change) at 1, 4, and 6 months [adjusted beta coefficient: 1 month 0.04 (95% CI 0.01, 0.06); 4 month 0.04 (95% CI 0.01, 0.06); and 6 month 0.04 (95% CI 0.00, 0.08)].

**Conclusion:**

Although week 1 LATCH and PIBBS scores were not associated with rates of exclusive breastfeeding, higher scores were positively associated with growth metrics among infants with LBW, suggesting that these tools may be useful to identify dyads who would benefit from early lactation support.

Breastfeeding is associated with many benefits for infants with low birth weight [LBW; birth weight (BW) < 2500 g (g)], a vulnerable population with a disproportionately high burden of both morbidities and mortality compared with normal BW infants particularly in low- and middle-income countries (LMICs).^1^^,^^2^ The increased health risks experienced by infants with LBW may be partly mitigated by the provision of human milk; however, the process of establishing breastfeeding is often difficult for this population.^3^^,^^4^

Breastfeeding assessment scales such as the Latch, Audible swallowing, Type of nipple, Comfort, and Hold (LATCH) and Preterm Infant Breastfeeding Behavior Scale (PIBBS) are designed to objectively quantify infant breastfeeding characteristics and competency. LATCH is an observational method used to systematically assess breastfeeding behaviors in term infants but has also been validated for use with infants born preterm.^5^^,^^6^ PIBBS was developed specifically for breastfeeding observations of infants born preterm.^7^ LATCH and PIBBS scales are two of the most widely studied breastfeeding assessment tools and have been validated in both high and low-middle income countries (HICs and LMICs)^5^^,^^8^, ^9^, ^10^, ^11^, ^12^, ^13^; however, most studies have been conducted in HICS. Also, nearly all studies in both HICs and LMICs have evaluated only hospitalized infants shortly after birth. To our knowledge, there have been no studies evaluating breastfeeding competency among discharged infants who were also LBW or correlating LATCH or PIBBS scores with longitudinal growth among infants with LBW.

The objective of this study was to describe breastfeeding competency scores through the first 6 weeks of age in a cohort of infants with moderately LBW in 3 LMIC settings and to determine the association between early breastfeeding competency scores and 2 key outcomes^1^: exclusive breastfeeding at 4- and 6-months and^2^ infant growth at 6 months.

## Methods

Breastfeeding observations were conducted as part of the Low Birthweight Infant Feeding Exploration (LIFE) study, an observational cohort study conducted across 12 hospitals at 4 sites: Karnataka State, India; Odisha State, India; Lilongwe, Malawi; and Dar es Salaam, Tanzania.^14^ Infants with LBW were enrolled from either the postpartum unit or the neonatal intensive care unit (ICU) within 72 hours of birth.

The LIFE study aim was to document current feeding practices and growth patterns among infants with LBW in LMICs.^14^ Key study results related to in-facility feeding practices and 6-month prospective cohort outcomes have been published elsewhere.^15^

Breastfeeding observations were conducted among infants with moderately LBW (BW 1500-2499 g) enrolled in the observational cohort of the LIFE study. Infants were enrolled within 72 hours of birth. We excluded infants with congenital anomalies that may affect feeding, severe neonatal encephalopathy, young mothers (under 18 years in Tanzania and India; 16–17 years and unmarried or under 16 years in Malawi per local regulatory guidelines), infants with a twin who died before 72 hours, and infants born to mothers who planned to move away from the catchment area. Enrolled infants who died before 72 hours were replaced and not included in the final data analysis.

Infants with LBW were included in the breastfeeding assessment subgroup only if they were observed to be breastfeeding during a routine study visit in the first 6 weeks after birth (weeks 1, 2, 4, or 6 of age) and the mother gave verbal permission to observe. The sample size of the breastfeeding cohort was determined by the number of infants among the 1,114 enrolled in the LIFE prospective cohort who were observed to be breastfeeding. For the parent study, we calculated the sample size for precision of estimates based on the percent of infants with LBW whose mean length-for-age z score (LAZ) at 6 months was <−2; with 300 dyads per site, we had precision of at least ±3.6% for a true proportion of 10% of infants.^14^

The study was registered with clinicaltrials.gov (NCT04002908) and Clinical Trial Registry of India (CTRI/2019/02/017475). This study was approved by 11 institutional review board (IRB) committees in India, Malawi, Tanzania, and the United States. All mothers who participated in the study provided written, informed consent at the time of study enrollment.

Baseline demographics and antenatal history were gathered at enrollment. All data collection tools were piloted prior to the initiation of the study, including breastfeeding assessments. We classified infants with LBW into 4 categories based on gestational age, determined using best obstetric estimate, and size-for-gestational age: preterm-appropriate-for-gestational-age (AGA), preterm-small-for-gestational-age (SGA), preterm-large-for-gestational-age (LGA), and term-SGA. We defined SGA as a BW < 10^th^ percentile for gestational age, AGA as a BW of 10-90^th^ percentile for gestational age, and LGA as a BW > 90^th^ percentile for gestational age per International Fetal and Newborn Growth Consortium for the 21^st^ Century (INTERGROWTH-21^st^) standards.^16^ Preterm was a gestational age < 37 weeks.

Breastfeeding assessments were conducted as direct observation of a breastfeeding session by trained study nurses or clinicians. Breastfeeding assessment training was led by a core team from Ariadne Labs that traveled to all sites. The LATCH and PIBBS trainings were provided by a pediatrician and consisted of lectures, videos, and standardization exercises. Study nurses completed the assessments during study visits when an infant was ready to be fed. The assessments were composed of questions from the LATCH and PIBBS tools. For LATCH, mother-infant dyads were scored on a 10-point scale,^6^ and for PIBBS, infants were scored on a 20-point scale.^7^

BW data were gathered by facility staff at birth. Study nurses conducted anthropometric assessments at enrollment and weeks 1, 2, 4, 18, and 26 in triplicate using standardized equipment (SECA 334 scale, SECA 417 infantometer, and Shorr tape). For infants born preterm, we used the INTERGROWTH-21^st^ newborn size at birth and preterm postnatal growth standards up to 6 months to determine Z-scores. For term infants, we used the WHO growth standards from birth to 6 months.^16^, ^17^, ^18^

We defined regaining BW as meeting or exceeding BW by the week 2 study visit. We defined slow week 2 weight gain as average weight gain under 20 g/day from weeks 1 - 2. We evaluated this metric during the second week after birth, a time in which infants are expected to regain BW after initial loss.^19^^,^^20^ We defined underweight as a weight-for-age z-score (WAZ) less than −2 and weight gain velocity as change in WAZ from baseline to a measured time point.

We report data on feeding patterns at 4 and 6 months of age. We defined exclusive breastfeeding as feeding only human milk at all data collection visits.^21^

### Data Analysis

We report descriptive statistics using means and standard deviations (SDs) or median and interquartile range (IQR) for continuous variables, and frequencies and percentages for categorical variables. Infants with missing data on any components included in either LATCH or PIBBS scales were not included in the results for that scoring system at that given time point. We used Pearson's correlation coefficient to assess the correlation between LATCH and PIBBS scores. We used unadjusted and adjusted regression to interrogate the association between LATCH and PIBBS scores and LBW type (linear regression), breastfeeding exclusivity (logistic regression), and growth (mixed effects models). We calculated both unadjusted and adjusted relative risks and beta coefficients for the association between early PIBBS score and certain growth metrics. Models were *a priori* adjusted for maternal age, parity, education, residence, delivery mode, LBW type, number of offspring, and site.

## Results

**Figure 1:**
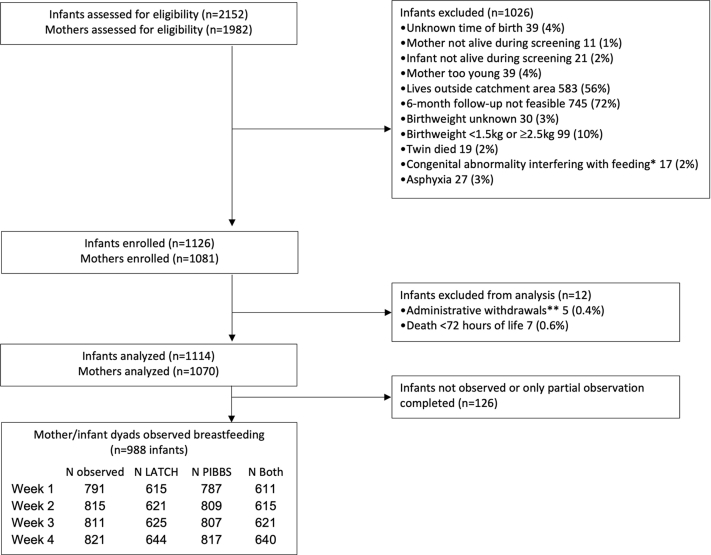
Study enrollment flow diagram. ∗Congenital abnormalities were limited to those interfering with feeding such as cleft lip or palate, hydrocephalus; gastrointestinal tract anomalies including gastroschisis, omphalocele or anal atresia; neural tube defects; congenital cardiac defects; suspected trisomy 21; suspected congenital infection. ∗∗Administrative withdrawals refer to infants who were withdrawn from the study by investigators in the first 72 hours because they were dually enrolled in a different research study at the same health facility.

We conducted data collection for the 6-month prospective cohort from September 13, 2019 until January 27, 2021. We screened 1,982 mothers and 2,152 infants for eligibility; 1070 mothers and 1114 infants were enrolled and analyzed (Figure 1, online; available at www.jpeds.com). We observed 988 mother-infant dyads breastfeeding during a study visit and completed a full LATCH or PIBBS assessment with 949 mother-infant dyads. We obtained anthropometric and/or feeding pattern data for 973 infants. Among 615 infants with completed week 1 LATCH scores, we obtained 6-month anthropometrics for 508 and were missing data for 107 due to loss-to-follow up or phone-based visits during the COVID-19 pandemic. Among 787 infants with completed week 1 PIBBS scores, we obtained 6-month anthropometrics on 688 and were missing data for 99.

Table I contains demographic information for mother-infant dyads for whom breastfeeding assessments were conducted. Slightly more than half of the infants were term-SGA (54.0%), with a higher percentage of term-SGA infants from the India sites compared with the Africa sites. The mean gestational age varied by LBW type [term-SGA: 38.6 weeks (SD ± 1.3); preterm-SGA: 35.8 weeks (SD ± 0.8); preterm-AGA: 34.3 weeks (SD ± 1.4); preterm-LGA infants: 29.7 weeks (SD ± 1.9)].

**Table I:** Demographics of Low Birthweight Infant Feeding Exploration (LIFE) cohort mothers and infants with LATCH/PIBBS scores

Maternal demographics	Tanzania	Malawi	India-Karnataka	India-Odisha	Total
N	291	250	283	125	949
Maternal age (yrs)	27 (5.9)	25 (6.2)	24 (3.9)	25 (4.3)	25.4 (5.3)
Secondary education or more	123 (42%)	112 (45%)	222 (78%)	59 (47%)	516 (54%)
Married	256 (88%)	231 (85%)	283 (94%)	125 (63%)	895 (94%)
Parity: 1	121 (42%)	92 (34%)	161 (54%)	72 (37%)	446 (42%)
Parity: 2	79 (27%)	54 (20%)	82 (27%)	43 (22%)	258 (27%)
Parity: 3+	91 (31%)	104 (38%)	40 (13%)	10 (5%)	245 (26%)
Previous LBW birth (n = 292)	13 (12%)	14 (18%)	40 (47%)	11 (55%)	78 (27%)
Maternal positive HIV status	14 (5%)	32 (12%)	0 (0%)	0 (0%)	46 (5%)
Feeding counseling at baseline	236 (80%)	237 (86%)	234 (81%)	63 (50%)	770 (78%)
Received antenatal care	290 (100%)	247 (99%)	282 (100%)	113 (90%)	932 (98%)
Antenatal care teaching					
Exclusive breastfeeding for 6 mos	165 (57%)	168 (68%)	111 (39%)	54 (48%)	498 (53%)
Breastfeeding position & attachment	137 (47%)	74 (30%)	18 (6%)	2 (2%)	231 (25%)
Expressing milk & storing milk	107 (37%)	24 (10%)	1 (0%)	0 (0%)	132 (14%)
Breastfeeding on Demand	133 (46%)	57 (23%)	3 (1%)	19 (17%)	212 (23%)
Benefits of breastfeeding	146 (50%)	100 (40%)	36 (13%)	23 (20%)	305 (33%)
Kangaroo mother care	92 (32%)	11 (4%)	0 (0%)	0 (0%)	103 (11%)

Infant Demographics					
N	297	275	291	125	988
Male sex	132 (44%)	131 (48%)	136 (47%)	48 (38%)	447 (45%)
Cesarean birth	106 (36%)	28 (10%)	138 (47%)	37 (30%)	309 (31%)
Birth weight (kg)	2.1 (0.3)	2.1 (0.2)	2.2 (0.2)	2.2 (0.2)	2.1 (0.24)
Gestational age (wks)	36 (3.1)	36 (2.6)	37 (2.1)	38 (1.9)	37 (2.7)
Term SGA	100 (34%)	132 (48%)	195 (67%)	105 (84%)	532 (54%)
Preterm SGA	53 (18%)	35 (13%)	39 (13%)	5 (4%)	132 (13%)
Preterm AGA	126 (43%)	93 (34%)	55 (19%)	14 (11%)	288 (29%)
Preterm LGA	17 (6%)	14 (5%)	2 (1%)	1 (1%)	34 (3%)

*HIV*, human immunodeficiency virus; *kg*, kilograms; *mos*, months; *wks*, weeks; *yrs*, years.

Continuous variables presented as mean (SD). Categorical variables presented as n (%).

We compared the 949 mother-infant dyads with at least 1 breastfeeding competency score (LATCH or PIBBS) to the 121 without a breastfeeding competency score. Most characteristics were similar, including maternal age, parity, antenatal care attendance, receipt of lactation support, and infant sex, BW, and gestational age. Mothers not observed to be breastfeeding during a study visit were slightly less likely to have attended secondary school or beyond (44% vs 54%) and were less likely to exclusively breastfeed at 6 months [not observed: 21% (15/72 infants with 6-month exclusive breastfeeding data) vs observed: 46% (411/901)].

**Figure 2:**
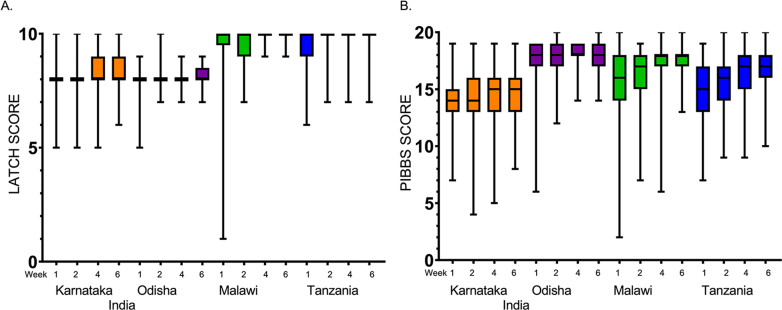
Boxplots of LATCH (A) and PIBBS (B) scores at weeks 1, 2,4, and 6 by study site.

Median LATCH score increased with time from a score of 8 (IQR 8,10; n = 615) at week 1 to 9 (IQR 8,10; n = 644) at week 6 (Figure 2, online; available at www.jpeds.com). Median PIBBS score similarly increased from 15 (IQR 13,17; n = 787) at week 1 to 17 (IQR 15,18; n = 817) at week 6. Median LATCH scores were higher at the Malawi and Tanzania sites than at the 2 India sites. PIBBS scores were lowest in Karnataka and highest in Odisha. Little variability was seen in LATCH scores at the given sites (Figure 2, online; available at www.jpeds.com). We found a weak, positive correlation between LATCH and PIBBS score at weeks 1, 2, 4, and 6 using the Pearson correlation coefficient [week 1: rho = 0.29, *P* < .001; week 2: 0.38, *P* < .001; week 4: rho = 0.39, *P* < .001; week 6: rho = 0.37, *P* < .001]. We analyzed the individual score items of each scale to identify the breastfeeding components that seemed to present the greatest challenge for infants with LBW in this cohort (Table II, online; available at www.jpeds.com). For LATCH, there was very little heterogeneity between score components. The components of the scale that were primarily mother-dependent (type of nipple, comfort, and requiring assistance with the hold) received nearly universally perfect scores. The lowest individual component scores were reported for *audible suckling*, which improved with time from a median week 1 score of zero to a median week 6 score of 2. PIBBS scores were notable for the improvement in nearly all components over time.

We compared LATCH and PIBBS scores between BW types (Table III, online; available at www.jpeds.com) There was no difference in LATCH or PIBBS scores when comparing BW types at any time point after adjusting for site.

Among infants with breastfeeding observations, 61% (560/908) were exclusively breastfed at 4 months and 46% (411/901) were exclusively breastfed up to 6 months. Neither LATCH nor PIBBS score at week 1 was associated with exclusive breastfeeding at 4- or 6- months on crude or adjusted analysis (Table IV). Unadjusted LATCH scores at weeks 2 and 4 were positively associated with exclusive breastfeeding at 4 months [week 2: RR 1.20 (95% CI 1.03, 1.41); week 4: RR 1.27 (1.07, 1.49)] and 6 months [week 2: RR 1.21 (95% CI 1.04, 1.41)]. This was not significant after adjustment.

**Table IV:** The association between week 1 continuous LATCH and PIBBS scores and exclusive breastfeeding at 4- and 6-months

Week 1	4 months			6 months		
	Exclusive breastfeeding	Crude RR (95% CI)	Adjusted RR (95% CI)	Exclusive breastfeeding	Crude RR (95% CI)	Adjusted RR (95% CI)
LATCH	356/568 (63%)	1.12 (0.96, 1.29)	1.09 (0.91, 1.31)	280/568 (49%)	1.08 (0.94, 1.25)	1.05 (0.89, 1.26)
PIBBS	455/727 (63%)	1.01 (0.95, 1.07)	1.00 (0.95, 1.06)	326/722 (45%)	1.00 (0.95, 1.06)	1.00 (0.95, 1.06)

Adjusted for maternal age, infant sex, maternal education, residence, delivery mode, number of offspring, LBW type, study site, and parity. The rates of exclusive breastfeeding are among infants with a week 1 LATCH or PIBBS score documented.

Twenty-two percent of infants (231/966) with a documented breastfeeding observation did not regain BW by 2 weeks of age. One-third (33%; 320/966) had a low growth velocity (<20 g/day) from birth to 2 weeks. Every additional point on the LATCH tool at week 1 was associated with a 38% higher likelihood of regaining BW by 2 weeks and 27% higher likelihood of average daily weight gain of at least 20 g/day by 2 weeks (Table V). Similarly, for each additional point on the PIBBS tool at week 1, infants were 16% more likely to regain their BW by 2 weeks and 6% more likely to have a growth velocity of greater than 20 g/day in the first 2 weeks of life. For every additional point on LATCH or PIBBS, there was a lower likelihood of being underweight at 6 months, though this association was not significant in the adjusted analysis (Table V).

**Table V:** The association between week 1 continuous LATCH and PIBBS scores and growth metrics

LATCH score at 1 week		Crude RR (95%CI)	Adjusted RR (95%CI)
Did not regain birth weight by 2 weeks	118	--	--
Did regain birth weight by 2 weeks	482	1.38 (1.15, 1.65)	1.42 (1.15, 1.76)
Slow growth velocity at 2 weeks (<20 g/day)	412	--	--
Velocity >20/day	188	1.22 (1.05, 1.42)	1.30 (1.08, 1.56)
Underweight at 6 months (WAZ < −2)	131	--	--
Not underweight at 6 months (WAZ ≥ −2)	377	1.06 (0.99, 1.12)	1.06 (0.99, 1.14)

PIBBS Score at 1 week	N	Crude RR (95%CI)	Adjusted RR (95%CI)
Did not regain birth weight by 2 weeks	145	--	--
Did regain birth weight by 2 weeks	625	1.15 (1.07, 1.22)	1.15 (1.07, 1.23)
Slow growth velocity at 2 weeks (<20 g/day)	533	--	--
Velocity >20/day	237	1.06 (1.00, 1.12)	0.96 (0.99, 1.12)
Underweight at 6 months (WAZ < −2)	194	--	--
Not underweight at 6 months (WAZ ≥ −2)	494	1.06 (0.99, 1.12)	1.06 (0.99, 1.14)

*WAZ*, weight-for-age Z-score.

Adjusted for maternal age, infant sex, maternal education, residence, delivery mode, birth count, LBW type, study site and parity. Growth velocity is measured as average daily weight changed between weeks 1 and 2.

**Figure 3:**
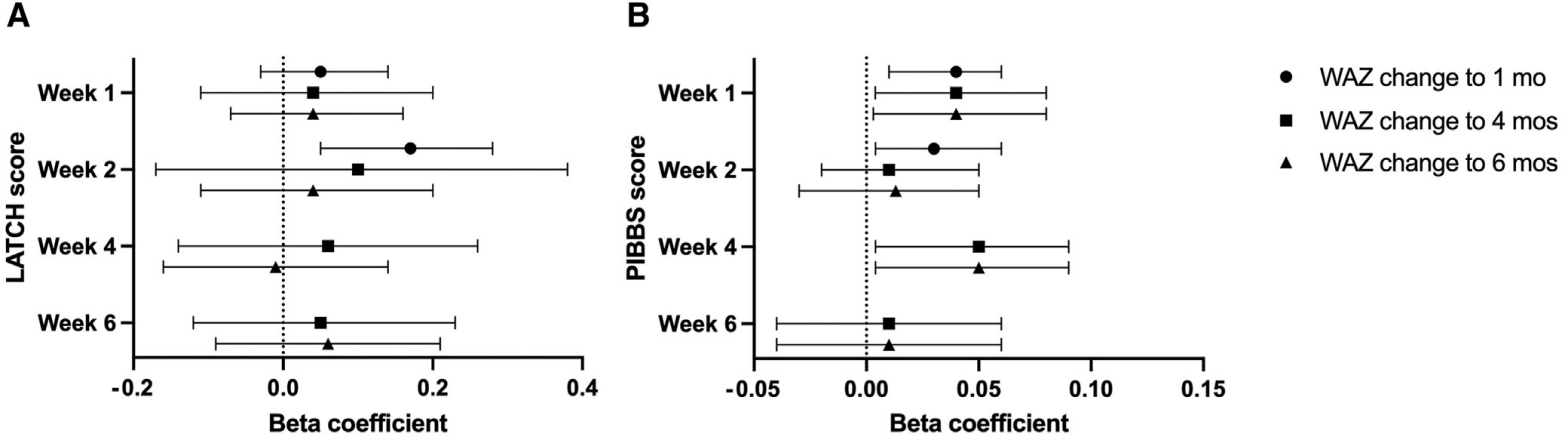
The association between LATCH and PIBBS scores and weight gain velocity (change in weight-for-age Z scores) from baseline to months 1, 4, and 6 shown as beta coefficients and 95% confidence intervals (CIs). Beta coefficients were adjusted for maternal age, infant sex, maternal education, residence, delivery mode, number of offspring, LBW type, site and parity. *WAZ*, weight-for-age Z score.

LATCH scores at weeks 1, 2, 4, and 6 were positively associated with weight gain velocity at 1, 4, and 6 months, though this trend was significant only when comparing the week 2 LATCH with the 1-month change in WAZ in the adjusted model [aRR 0.77 (95% CI 0.60, 0.97)] (Figure 3A). PIBBS scores showed a positive trend in weight gain velocity at every time point in the adjusted model (Figure 3B). Every additional point on the PIBBS score at week 1 was associated with an increase in +0.04 WAZ scores at 1, 4, and 6 months [adjusted beta coefficient (95% CI): 1 month 0.04 (0.01, 0.06); 4 month 0.04 (0.01, 0.06); 6 month 0.04 (0.00, 0.08)]. Similarly, PIBBS scores at week 4 were positively associated with change in WAZ from birth to 4 and 6 months.

## Discussion

LATCH and PIBBS tools are a means of assessing breastfeeding competency among infants with LBW. These tools have been used and validated among infants in LMICs including India and sub-Saharan Africa.^8^^,^^10^^,^^11^^,^^22^^,^^23^ In this cohort of infants with LBW, improved early breastfeeding competency, as measured by a higher LATCH or PIBBS score, was associated with improved growth in early infancy. We found that breastfeeding competency improved throughout the first 6 postnatal weeks for the infants with LBW enrolled in this multi-country cohort, particularly the domains related to infant stamina. These tools may have a valuable clinical application to identify infants with risk factors for poor growth who would most benefit from targeted lactation interventions.

We did not find an association between LBW type and breastfeeding competency among infants in this cohort. Although median LATCH scores were slightly lower among the term SGA group compared with infants born preterm who were AGA, AGA, or SGA, adjusted linear regression analysis demonstrated no association between LBW type and LATCH or PIBBS score. Previously, PIBBS scores were found to be lower for infants born preterm compared with infants born at term in a validation study conducted among hospitalized infants in Sweden.^7^ Similarly, median LATCH scores were lower in infants born preterm compared with term in a neonatal intensive care unit in Turkey, an upper middle income setting.^5^ Interestingly, a single study of PIBBS among infants born preterm in Sweden found lower gestational age to be associated with earlier evidence of breastfeeding competency. This comparison was different than ours as it compared infants with a similar corrected postmenstrual age, whereas we compared infants with a similar postnatal age.^24^ Our study is unique in that most other studies have compared infants born preterm to infants born at term who were AGA, not infants born at term who were SGA. Our findings suggest that full-term infants who are SGA with LBW may struggle with early breastfeeding in a manner similar to infants born preterm. Due to their full-term status, these infants are often discharged rapidly after birth and may not receive the dedicated attention to feeding support that our findings suggest that this population may warrant. These infants may benefit from specialized lactation support.

With regard to early breastfeeding competency and prolonged breastfeeding exclusivity, neither LATCH nor PIBBS scores were associated with exclusive breastfeeding at 4- or 6-months among this cohort, suggesting that postdischarge breastfeeding competency may not be a critical driver in exclusive breastfeeding up to 6 months. This is a bit surprising as previous literature has suggested that improved early breastfeeding competency is associated with breastfeeding exclusivity, but this association has been largely limited to 6-week breastfeeding patterns. For LATCH, there have been several studies correlating early breastfeeding competency with 6-week breastfeeding exclusivity, including 3 studies based in India among term or near-term infants.^25^, ^26^, ^27^ All studies demonstrated a positive association between LATCH score and exclusive breastfeeding up to 6 weeks, but no studies reported the association between early LATCH score and 6 months breastfeeding exclusivity.^25^, ^26^, ^27^, ^28^, ^29^ We found no previous studies examining the association between PIBBS and exclusive breastfeeding. These studies also differ from ours in that they evaluated healthy, full-term or nearly full-term infants. The infants with LBW in our cohort are a unique sample with specific vulnerabilities aside from early breastfeeding competency that may have determined their likelihood of exclusive breastfeeding at 4- and 6-months.

Most studies of breastfeeding competency and growth have been limited to early growth metrics; however, we found that higher LATCH and PIBBS scores were associated with improved growth up to 6 months among infants with LBW in our cohort. We found a general positive association between LATCH and/or PIBBS and growth at multiple time points, including BW regain and weight gain velocity at 2 weeks and 1, 4, and 6 months. These findings have potentially important clinical implications, as LATCH or PIBBS may be a valuable tool to identify infants at risk for poor early growth. These tools could be used either in patients or in the community by community-based health workers during the assessment of infants with LBW. Very few other studies have examined the relationship between breastfeeding observational scores and growth. Two studies of healthy, predominantly full-term infants in India and Turkey have found that infants with higher LATCH scores were less likely to have excessive weight loss of greater than 10% of BW than those with lower LATCH scores.^5^^,^^23^ Another study of full-term infants in India found LATCH scores greater than or equal to 6 to be associated with a higher likelihood of gaining more than 20 g/day at 6 weeks of age.^27^ We found no previous studies that reported the association of LATCH or PIBBS scores and growth up to 6 months of age, but our positive findings suggest that the metric of breastfeeding competency scores may be useful for early identification of infants with LBW at higher risk for poor growth in later infancy.

A strength of the study is the novel association between early feeding competency and later growth. Additionally, there have been little to no data published regarding direct breastfeeding observations of infants with LBW in LMICs. Even in high income settings, most breastfeeding observational data only account for breastfeeding characteristics within the first few days after birth, whereas we collected LATCH and PIBBS data up to 6 weeks of age. Our findings were strengthened by the robust feeding and growth data collected as part of the study and the multicountry nature of our cohort.

Our study has limitations related to LATCH and PIBBS. Observations were based on a convenience sample of infants for whom a breastfeeding session overlapped with a follow-up visit. Nearly 90% of infants were observed, so our findings are representative of most infants with LBW in this cohort, but the infants not observed were more likely to have mothers without a secondary education and less likely to be exclusively breastfeeding at 4 or 6 months of age. For future studies, additional efforts could be made to coordinate data collection with a breastfeeding session, such as timing study visits around expected feeding times or prolonging visits to overlap with a feed. Another challenge was the loss of follow up and missing data related to restrictions in data collection during the COVID-19 pandemic. We tried to minimize this by extending study visit windows or conducting phone interviews when in-person visits were not possible.

Future research could focus on the implementation and acceptability of the LATCH and PIBBS tools within the health system as potentially valuable tools to identify infants at risk for breastfeeding difficulty and poor growth.

The results of our study showed that LATCH and PIBBS scales are 2 tools to assess breastfeeding competency which may have a direct clinical application for identifying high-risk infants who could benefit from targeted lactation support. These tools may be particularly useful for health workers to prioritize limited lactation resources among mother/infant dyads in resource-constrained settings. In this novel analysis of the association between early breastfeeding competency and subsequent exclusive breastfeeding and infant growth, LATCH and PIBBS scores were associated with multiple key growth metrics. Our findings have direct clinical relevance for practitioners caring for infants with LBW, particularly in low-resource settings. LATCH and PIBBS are tools that could be used by clinicians to identify infants at increased risk of poor breastfeeding and poor growth, who would benefit from targeted lactation interventions and closer growth monitoring. Additionally, LATCH and PIBBS may be useful as outcome indicators for breastfeeding and lactation support interventions.

Ethics approval and consent to participate: This study was approved by 11 ethics committees in India, Malawi, Tanzania and the USA.

(1) India Health Ministry's Screening Committee (Indian Council of Medical Research acting as its secretariat) (2019–2674)
(2) Directorate of Health and Family Welfare Services, Government of Karnataka including Women and Children Hospital, Davangere and Chigateri General District Hospital, Davangere (NHM/SPM/04/2019–20)
(3) KLE Academy of Higher Education and Research Institutional Ethics Committee which also covers investigators at JN Medical College, Belagavi and KLES Dr Prabhakar Kore Hospital & Medical Research Center, Belagavi (KAHER/IEC/2019-20/D-2760)
(4) SS Institute of Medical Sciences & Research Centre Institutional Ethics Review Board (IERB/200/2019)
(5) JJM Medical College Institutional Ethics Committee (JJMMC/IEC-01/2019) which also covers investigators at Bapuji Child Health Institute & Research Centre, Davangere, Women & Children Hospital, Davangere and Chigateri General District Hospital, Davangere
(6) Directorate of Health Services Research and Ethics Committee, Odisha State/City Hospital Oriya Bazar, Cuttack (155/PMU/187/17)
(7) Sriram Chandra Bhanja Medical College Institutional Ethical Committee, Cuttack (7188)
(8) Malawi National Health Sciences Research Committee (NHSRC2019/Protocol19/03/2250-UNCPM 21905)
(9) Tanzania National Institute of Medical Research (NIMR/HQ/R.8a/Vol.IX/3126)
(10) Muhimbili University of Health and Allied Sciences (DA.282/298/01.C/)
(11) Harvard T.H Chan School of Public Health (IRB10-0282) - covers investigators at Boston Children's Hospital, Brigham and Women's Hospital, Emory University, PATH, and University of North Carolina.

For the prospective cohort enrollment, women provided written informed consent.

## CRediT authorship contribution statement

**Krysten North:** Conceptualization, Methodology, Writing – original draft, Writing – review & editing. **Katherine E.A. Semrau:** Conceptualization, Data curation, Formal analysis, Funding acquisition, Investigation, Methodology, Supervision, Writing – original draft, Writing – review & editing. **Roopa M. Bellad:** Conceptualization, Data curation, Formal analysis, Investigation, Methodology, Resources, Supervision, Writing – review & editing. **Sangappa M. Dhaded:** Conceptualization, Data curation, Formal analysis, Investigation, Methodology, Resources, Supervision, Writing – review & editing. **Leena Das:** Investigation, Supervision, Writing – review & editing. **Jnanindranath N. Behera:** Investigation, Supervision, Writing – review & editing. **Irving Hoffman:** Investigation, Writing – review & editing. **Tisungane Mvalo:** Data curation, Supervision, Writing – review & editing. **Rodrick Kisenge:** Methodology, Supervision, Writing – review & editing. **Christopher R. Sudfeld:** Data curation, Methodology, Resources, Supervision, Writing – review & editing. **Sarah Somji:** Investigation, Supervision, Writing – review & editing. **Rana R. Mokhtar:** Data curation, Formal analysis, Investigation, Supervision, Visualization, Writing – review & editing. **Linda Vesel:** Conceptualization, Data curation, Methodology, Writing – review & editing. **Shivaprasad Goudar:** Conceptualization, Investigation, Supervision, Writing – review & editing. **Sunil S. Vernekar:** Conceptualization, Data curation, Formal analysis, Investigation, Methodology, Resources, Supervision, Writing – review & editing. **E.S. Siddhartha:** Investigation, Supervision, Writing – review & editing. **Bipsa Singh:** Investigation, Supervision, Writing – review & editing. **M.B. Koujalagi:** Investigation, Supervision, Writing – review & editing. **Sanghamitra Panda:** Investigation, Supervision, Writing – review & editing. **Eddah Kafansiyanji:** Data curation, Writing – review & editing. **Naomie Nyirenda:** Data curation, Writing – review & editing. **Melda Phiri:** Data curation, Writing – review & editing. **Friday Saidi:** Data curation, Supervision, Writing – review & editing. **Nahya S. Masoud:** Methodology, Supervision, Writing – review & editing. **Robert Moshiro:** Methodology, Supervision, Writing – review & editing. **Danielle E. Tuller:** Conceptualization, Funding acquisition, Methodology, Supervision, Writing – review & editing. **Kiersten Israel-Ballard:** Conceptualization, Methodology, Writing – review & editing. **Christopher P. Duggan:** Conceptualization, Methodology, Writing – review & editing. **Anne C.C. Lee:** Methodology, Supervision, Writing – review & editing. **Kimberly L. Mansen:** Conceptualization, Methodology, Writing – review & editing. **Melissa F. Young:** Conceptualization, Methodology, Writing – review & editing. **Karim Manji:** Conceptualization, Investigation, Methodology, Supervision, Writing – review & editing.

## Declaration of Competing Interest

Conflict of interest: The Bill and Melinda Gates Foundation reviewed the study design, but had no role in data collection, management, analysis, interpretation, writing of the manuscript, or the decision to submit manuscripts for publication. This study was registered at the following: Clinicaltrials.gov (NCT04002908) and the Clinical Trial Registry of India (CTRI/2019/02/017475, http://ctri.nic.in). The authors have no conflicts of interest relative to this study to affirm. Co-author Dr. Christopher Duggan is an editor at *The Journal of Pediatrics* but was not involved in the editorial assessment of this manuscript.

Funding: This work was supported, in whole or in part, by the 10.13039/100000865Bill & Melinda Gates Foundation, grant number OPP1192260. Under the grant conditions of the Foundation, a Creative Commons Attribution 4.0 Generic License has already been assigned to the Author Accepted Manuscript version that might arise from this submission. CPD was supported in part by P30 DK040561. 10.13039/100006307ACL was supported in part by 5K23HD091390.

The first draft of this manuscript was written by Krysten North. No person received an honorarium, grant, or other form of payment to produce the manuscript.

The authors declare no conflicts of interest.

## Supplementary Data

STROBE_checklist

Table II

Table III
