# Supplementary material for: The Association Between Breastfeeding and Growth Among Infants with Moderately Low Birth Weight: A Prospective Cohort Study
Source: J Pediatr. 2024 Jun;269:114003. doi: 10.1016/j.jpeds.2024.114003 (PMC11155439; doi:10.1016/j.jpeds.2024.114003)
Supplement: Table II [file mmc2.docx]

| Table 2. Median and interquartile range of individual LATCH and PIBBS score components by observation week. | | | | | | | | | | |  |  |
| --- | --- | --- | --- | --- | --- | --- | --- | --- | --- | --- | --- | --- |
| **LATCH** | | | | |  | **PIBBS** | | | | | |  |
| **Component**  Median (IQR) | **1 week** | **2 week** | **4 week** | **6 week** |  | **Component**  Median (IQR) | **1 week** | **2 week** | **4 week** | **6 week** | | |
|  | *n=615* | *n=621* | *n=625* | *n=644* |  |  | *n=787* | *n=809* | *n=807* | *n=817* | | |
| **Latch** | 2 (2,2) | 2 (2,2) | 2 (2,2) | 2 (2,2) |  | **Rooting** | 2 (1,2) | 2 (1,2) | 2 (2,2) | 2 (2,2) | | |
| **Audible Suckling** | 0 (0,2) | 1 (0,2) | 1 (0,2) | 2 (0,2) |  | **Latch** | 3 (3,3) | 3 (3,3) | 3 (3,3) | 3 (3,3) | | |
| **Type of Nipple** | 2 (2,2) | 2 (2,2) | 2 (2,2) | 2 (2,2) |  | **Staying Latched** | 1 (1,2) | 1 (1,2) | 1 (1,2) | 1 (1,2) | | |
| **Comfort** | 2 (2,2) | 2 (2,2) | 2 (2,2) | 2 (2,2) |  | **Sucking** | 4 (3,4) | 4 (3,4) | 4 (4,4) | 4 (4,4) | | |
| **Hold** | 2 (2,2) | 2 (2,2) | 2 (2,2) | 2 (2,2) |  | **Long Sucking Burst** | 4 (2,5) | 4 (2,6) | 4 (3,6) | 5 (3,6) | | |
|  |  |  |  |  |  | **Swallowing** | 2 (1,2) | 2 (2,2) | 2 (2,2) | 2 (2,2) | | |
| **Total score** | 8 (8,10) | 9 (8,10) | 9 (8,10) | 10 (8,10) |  | **Total score** | 15 (2.6) | 15.6 (2.4) | 16.1 (2.3) | 16.4 (2.1) | | |
