# Supplementary material for: The Association Between Breastfeeding and Growth Among Infants with Moderately Low Birth Weight: A Prospective Cohort Study
Source: J Pediatr. 2024 Jun;269:114003. doi: 10.1016/j.jpeds.2024.114003 (PMC11155439; doi:10.1016/j.jpeds.2024.114003)
Supplement: Table III [file mmc3.docx]

**Table 3: Median and interquartile range of PIBBS/LATCH scores by LBW type.**

| **LATCH** | **1 week** | | **2 weeks** | | **4 weeks** | | **6 weeks** | |
| --- | --- | --- | --- | --- | --- | --- | --- | --- |
|  | n | median (IQR) | n | median (IQR) | **n** | median (IQR) | **n** | median (IQR) |
| Term SGA | 357 | 8 (8,10) | 338 | 8 (8,10) | 342 | 9 (8,10) | 346 | 9 (8,10) |
| Preterm SGA | 81 | 10 (8,10) | 79 | 10 (8,10) | 83 | 10 (8,10) | 92 | 10 (8,10) |
| Preterm AGA | 158 | 10 (8,10) | 158 | 10 (8,10) | 160 | 10 (8,10) | 187 | 10 (9,10) |
| Preterm LGA | 18 | 8.5 (8,10) | 19 | 10 (9,10) | 18 | 10 (10,10) | 19 | 10 (10,10) |
| **p-value*** |  | 0.23 |  | 0.87 |  | 0.45 |  | 0.801 |
|  |  |  |  |  |  |  |  |  |
| **PIBBS** | **1 week** | | **2 weeks** | | **4 weeks** | | **6 weeks** | |
|  | n | median (IQR) | n | median (IQR) | **n** | median (IQR) | **n** | median (IQR) |
| Term SGA | 437 | 15 (14.17) | 429 | 16 (14, 18) | 430 | 16 (14,18) | 427 | 17 (15, 18) |
| Preterm SGA | 102 | 15 (13,17) | 104 | 16 (14,17) | 104 | 16 (15, 18) | 113 | 17 (15, 18) |
| Preterm AGA | 220 | 15 (13.5, 16) | 215 | 16 (14,18) | 215 | 17 (15, 18) | 245 | 17 (16, 18) |
| Preterm LGA | 27 | 14 (13.17) | 29 | 16 (14,17) | 29 | 16 (15,18) | 31 | 17 (15, 18) |
| **p-value*** |  | 0.38 |  | 0.85 |  | 0.15 |  | 0.65 |

*****Type III p-value for the test of the composite null hypothesis that all levels of LBW type have the same feeding scores adjusted for study site.
